# War and peace in public health education and training: a scoping review

**DOI:** 10.1186/s12889-024-19788-w

**Published:** 2024-08-24

**Authors:** Lisa Wandschneider, Anna Nowak, Marta Miller, Anina Grün, Yudit Namer, Tomasz Bochenek, Lukasz Balwicki, Oliver Razum, Colette Cunningham

**Affiliations:** 1 Department of Epidemiology and International Public Health, School of Public Health, Bielefeld University, Bielefeld, Germany; 2 Research Institute Social Cohesion, Bielefeld University, Bielefeld, Germany; 3 School of Public Health, University College Cork, Cork, Ireland; 4 Medical University of Gdansk, Gdanks, Poland; 5 Department of Psychology, Health & Technology, Faculty of Behavioural, Management and Social Sciences, University of Twente, Enschede, Netherlands; 6 Department of Nutrition and Drug Research, Institute of Public Health, Faculty of Health Sciences, Jagiellonian University Medical College, Krakow, Poland; 7 Department of Public Health and Social Medicine, Medical University of Gdansk, Gdansk, Poland

**Keywords:** War, Prevention of war, Peace promotion, Public health education, Graduate education, Training, Workforce development, Curriculum, Europe

## Abstract

**Background:**

Armed conflict and war are public health disasters. Public health action has a crucial role in conflict-related emergencies and rehabilitation but also in war prevention and peace promotion. Translating this into public health training and competencies has just started to emerge, especially in Europe.

**Methods:**

We conducted a Scoping Review to map and identify the role of public health education and training of public health workforce relating to the prevention of war and promoting peace, as reflected in the scientific literature. We searched in PubMed, CINAHL, PsycINFO, Embase, Web of Science Core Collections as well as the reference list of included material in English, German and Polish. Focusing initially on the European region, we later expanded the search outside of Europe.

**Results:**

We included 7 publications from opinion pieces to an empirical assessment of curricula and training. The educational programs were predominantly short-term and extra-curricular in postgraduate courses addressing both public health professionals in conflict-affected countries as well as countries not directly affected by war. Publications focused on public health action in times of war, without specifying the context and type of war or armed conflict. Competencies taught focused on emergency response and multi-disciplinary collaboration during emergencies, frequently drawing on experience and examples from natural disaster and disease outbreak management.

**Conclusions:**

The scientific discourse on competences in public health education for times of war and for the promotion of peace, predominately focuses on immediate emergency response actions. The prevention of war and the promotion of peace are missing foci, that need to feature more prominently in public health training. Public Health Education and training should ensure that war prevention and peace promotion, as well as public health action in times of war, are included in their competencies for public health professionals.

**Supplementary Information:**

The online version contains supplementary material available at 10.1186/s12889-024-19788-w.

## Introduction

### War has profound adverse effects on public health [1]

War and armed conflict have far-reaching consequences, affecting the lives of millions of people, resulting in the loss of human life, but also in the disruption of social infrastructure such as safe food and water supplies, housing, and access to health services, leading to increased mortality and morbidity. War-torn countries and populations experience an increase in Disability Adjusted Life Years (DALYs) lost [2]. In addition, mass displacement, which disproportionately affects women, children, the elderly and vulnerable groups, exposes people to precarious living conditions and arduous travel, affecting their health and well-being. War and armed conflict have profound long-term physical and psychological consequences for those involved. They destroy communities and the fabric of society is often irreversibly undermined. This affects public health as well as legalizing and promoting violence as a way of solving problems [2]. In this paper, war and armed conflict is defined as ‘hostile contention by means of armed forces, carried on between nations, states, or rulers, or between parties in the same nation or state; the employment of armed forces against a foreign power, or against an opposing party in the state [3]. The International Committee of Red Cross (ICRC) casebook differentiates between an international armed conflict which “occurs when one or more States have recourse to armed force against another State, regardless of the reasons or the intensity of this confrontation” and a non-international armed conflict in which one or more non-State armed groups are involved (the vast majority of conflicts since 1945) [4]. Other authors differentiate wars by causes or intentions, for example imperial wars [5] or wars of annexation, such as Russia’s invasion of Ukraine. The reason is that preventive measures may be quite different. Much of the literature such as Levy et al. [1, 6] covers armed conflict but not wars of annexation [2, 7]. Today’s wars are often hybrid, meaning they are being fought by military as well as by destabilizing, non-military means [8]. Debates on preventing wars of annexation need to cover the role of the military, and the way in which public health relates to it, but also political determinants. For reasons of readability, however, the term ‘war’ will be used consistently throughout the text to include armed conflict and wars of annexation, as well as hybrid elements of warfare.

Conceptualizing war in public health is a relatively recent development, while international relations and defense studies have a longstanding tradition of analyzing and theorizing war and peace (e.g. [5, 9, 10]). Public health approaches focusing primarily on emergency response and relief care [11–13]. However, there is a growing shift in public health to emphasize its preventive role in peacetime, with the aim of minimizing the health impact of war on affected populations [14–16]. At the same time, one of public health’s major aims – reducing and dismantling (health) inequities — is also a crucial determinant for preventing armed conflict [17–19] presenting another valuable angle for public health’s role in peacebuilding and preventing war. This shift is consistent with modern public health care, given the emergence of complex global public health crises such as climate change, biodiversity loss, migration, cybersecurity, inequities, and pandemics such as COVID-19 [20]. We are looking at syndemics, whereby a set of linked health problems interact synergistically and contribute to the excess burden of disease in a population [21]. The preventative role provides an opportunity to develop a comprehensive public health framework that can effectively address the needs during war, its prevention, and post-conflict periods. Hagopian and Jabbour (2022) [22] proposed such a framework, using the Primary, Secondary and Tertiary Prevention (PSTP) Framework to address global inequalities and injustices that may contribute to war. It includes primary prevention, which focuses on addressing the root causes and social determinants of war; secondary prevention, which aims to reduce harm and damage during conflict; and tertiary prevention, which involves rebuilding health systems in post-conflict settings [22]. Wars of annexation have more to do with (re-)building imperia, rather than with inequalities and injustices. Nonetheless, there is no agreed-upon theory on the cause(s) of war [10].

While the role of public health in emergencies is increasingly well explored in public health research and in interdisciplinary collaboration, the translation of this knowledge into public health training and competencies is only just beginning to emerge, particularly in Europe. The literature is increasingly recommending that public health education and training programs should include an understanding of the health impacts of war and conflict, including the epidemiology of war-related injuries and illnesses, environmental impacts of war, and mental health effects of war on soldiers and civilians [2, 23–25]. Additionally, public health professionals should be trained to respond to the health impacts of war, including strategies for preventing and treating war-related injuries and illnesses, as well as promoting peace and non-violent conflict resolution [2, 23–25].^,.^

In light of contemporary conflicts and geopolitical tensions, such as the war in Ukraine, as well as those in Syria, Yemen, Myanmar, Israel and Gaza, and other regions, training on effective and systematic public health practice to support affected populations is needed. The role of public health in both the prevention of war and the promotion of peace work is increasingly recognized and calls for its inclusion in public health education, and in the training of public health professionals [7, 25, 26]. Considering the present circumstances, there is a need for public health professionals to develop a skill set that enables them to address the challenges of war, prevention, as well as peacebuilding, so that they can confidently navigate an increasingly multi-disciplinary role and take an active place in the dialogue on the prevention of war and its consequences. We conducted a Scoping Review of the scientific literature in public health to map available evidence and discourse on war. We covered public health perspectives on war prevention, reaction to war, rehabilitation, and peace promotion within public health education and training. More specifically we aimed to answer the following research questions:

- How is public health education addressing competencies related to war and peacebuilding? Are there existing examples of teaching and can we build on them for future training?
- What are the gaps in public health education on war and how can we address them?
- Are there existent frameworks which can be used or adapted to develop public health education programs focused on war, war prevention, and peace promotion?

## Methods

The research team, representing expertise from schools of public health across Europe, developed the search strategy with the help of the Population, Comparison and Context (PCC) Framework [27]. We opted for a Scoping Review methodology, since it allows for ‘a preliminary assessment of potential size and scope of available research literature’ as well as ‘identify the nature and extent of research evidence’ [28, 29]. It enabled us to map a diverse range of evidence e.g., implementation research on training programs as well as commentaries and opinion pieces, as indicators of an on-going discourse within the public health community. War and peacebuilding in public health education represent an under-researched area, therefore our Scoping Review allowed for the capture and extent, as well as the type of available evidence. Our methodology was developed using the PRISMA-ScR Checklist to comply with reporting and methodological standards (supplementary material 1) [30]. A review protocol was not published, but the authors used an internal methods protocol which has been updated throughout the process (Supplementary material 3).

### Search strategy

We searched in academic databases PubMed, CINAHL, PsycINFO, Embase, Web of Science Core Collections (using keywords and MeSH terms) to explore the scientific discourse on war and peacebuilding in public health education. Exploring grey literature, such as the extent of material at the level of Schools of Public Health exceeded the scope of this review and requires additional data collection tools. As related fields like disaster management and preparedness in public health are well represented within the scientific discourse, limiting this first mapping to scientific databases only, allows for a direct comparison and therefore seemed reasonable.

In our review, we defined the population as the body of interest, i.e., education and teaching body. Our search terms were chosen to identify literature that focused on public health education and training on war, war prevention and peace promotion. This method allowed the authors to capture examples of a broad range of education programs and training, both from individual courses to curricula. The concept element represents the thematic focus on war and peacebuilding. We aimed to integrate different stages of war e.g., armed conflict, active war and peacebuilding. In addition, we linked these search terms with closely-related fields, such as disaster management and preparedness, especially as these are competencies that are frequently linked to the context of war [31]. These terms have been informed by conflict-related health research as well as conceptual models systematizing the impact of war and peace promotion on public health [11–13, 15, 16]. For the context, being the third and last element of the PCC Framework, we used public health as the discipline and added global health since this is a field where the topic of war is frequently documented and addressed.

An experienced librarian supported the development of the search strategy (Table 1, search protocols for all databases can be found in supplementary material 2). We conducted the searches on 8th September 2022 and extracted the records into the reference manager Zotero. In addition to the search in scientific databases, we checked the reference lists of included sources for further records that could warrant inclusion.

**Table 1:** Search strategy in PubMed

**PCC element** (linked by AND)	**Search string** Filter: Title/Abstract
Population	“teaching” OR “education” OR “training” OR “course” OR “classroom” OR “workforce development” OR “capacity building” OR “competence” OR “competencies” OR “curriculum” OR “curricula” OR „syllabus “ OR “syllabi “ OR “pedagogy “ OR “pedagogic “ OR “toolkit” OR “schools of public health” OR “school of public health “ OR “public health department” OR “public health faculty”
Concept	“war” OR “armed conflict” OR “mass violence” OR “ “warfare” OR “combat “ OR “military” OR “peace “ OR “peace promotion” OR “peacebuilding” OR “peace building” OR “disaster management” OR “emergency response” OR “emergency preparedness” OR “conflict response “ OR “disaster recovery” OR “humanitarian crisis”
Context	“Public health” OR “global health”

### Eligibility criteria and study selection

In our review, we only included sources that had a primary interest in war and peacebuilding in the public health education context. This included publications that identified curricula, workshops, competencies, skills-sets and capacity-building trainings. For the war and peacebuilding element, this required an explicit definition of war and/or peacebuilding as the field of action or interest. We included academic literature, ranging from peer-reviewed articles to commentaries and editorials to capture a broad spectrum of the scientific discourse. We did not exclude any publication based on study design or period of the study.

We excluded sources that mentioned war or peacebuilding but did not elaborate further on how the courses addressed the specific needs or circumstances. Also, any material that solely addressed terrorist attacks was considered ineligible despite representing a potential weapon or strategy of war. Terrorist attacks were considered ineligible because they pertained to one, timely limited event that usually did not destroy the infrastructure of a complete region or country. Other competencies and frameworks for public health professionals is therefore warranted. The scoping review languages of the potential sources was limited to English, German, Polish.

For the screening process, we conducted a pilot with a random sample of 5% of the total records. This ensured inter-rater reliability between the reviewers by detecting inconsistencies and allowed us to adapt the eligibility criteria accordingly. Then, the reviewers (LW, MM, CC) started with the title and abstract screening. For the full texts, the reviewers changed (LW, MM, AG, AN), so we again conducted a pilot of a random 5% sample of the records identified in the abstract and title screening. Disagreements on the eligibility of full texts were discussed with all reviewers (LW, MM, AG, AN, CC) and resolved through discussion.

### Data charting and analysis

The data charting and extraction focused on the context of war and the education/teaching element of the material, rather than the study details. The data charting table includes basic study characteristics, information on the context of war and peacebuilding as well as the teaching intervention **(**Table 2). The standardized form guided the data charting process and was also tested in another pilot round between the reviewers. LW, MM, AG and AN extracted the data and modified the charting form in an iterative process, which was then discussed with the core reviewer team LW, MM, AG, AN and CC.

**Table 2:** Data extraction template

Category	Description
Reference	Full reference of the original article
Type of material	Study, commentary/editorial, dissertation, book chapter
Study design	Qualitative, quantitative or mixed-methods study, plus more detailed information on the study (e.g., cross-sectional study, focus groups)
Geographic origin of the material	For studies: the country/countries where the study has been conducted for commentaries/opinion pieces: list all countries identified by the authors’ affiliations
Aims	Aims as stated by the authors
Key topic	Overarching topics might include: • Health care system level • Legal dimension of war (international conventions, law, types of war) • “Vulnerable” populations (children, women, LGBTQI + , displaced people, detainees, war veterans) • Prevention of war & Peace building work
Discipline of public health	e.g., epidemiology, global health, public health law
Related disciplines/collaborations	e.g., military forces
Type of war/armed conflict	• Civil war, cross border war, armed conflict, • Geographical setting: where does the war/armed conflict take place? • Periodic reference • Which phase of war is addressed in the material? Early warning/prevention and preparedness, conflict, emergency, recovery/rehabilitation.^11,20^
**Teaching material**	
Brief description of training	What is this training about? How has this been developed? Who was part of the process developing and implementing it?
Level of Programme	Bachelors, Masters, PhD
Type of Programme	Integration in curricula or add-on material? Mandatory or selective courses?
Level of development	Development or presentation of teaching material, implementation, or evaluation research?
Availability of the material	Is the teaching material publicly available?

The data charting form also guided the descriptive and narrative synthesis of the findings. To structure the narrative analysis, we categorized the findings by the phase of war differentiating between a) preparedness and prevention, b) ongoing conflict and emergency, as well as c) recovery and rehabilitation based on Hagopian and Jabbour’s framework [22].

## Results

**Fig. 1:**
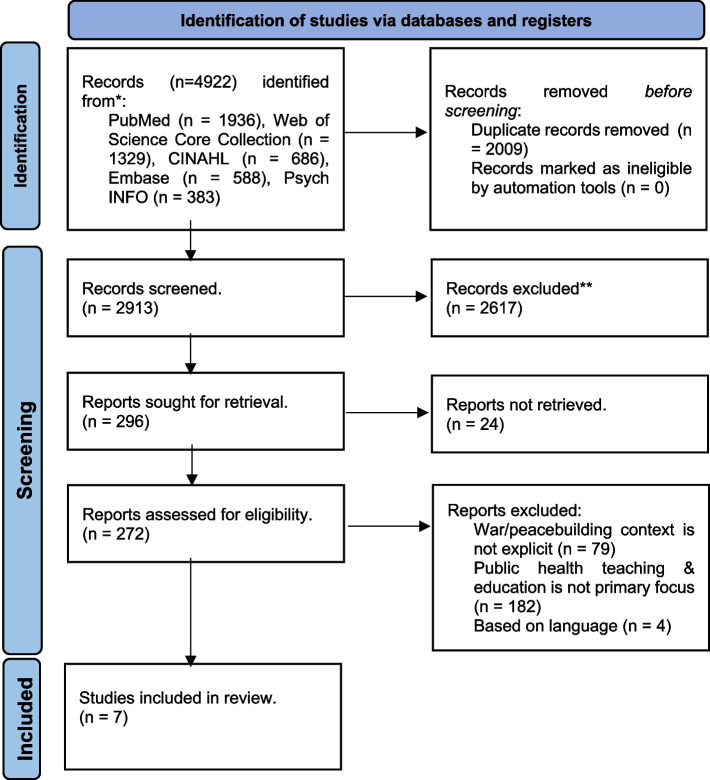
Flowchart on the process identifying studies. This Study Flow Chart details the flow of information throughout the distinct phases of the review: identification, screening and included studies for final review

The initial literature search resulted in a total of 4922 citations (Fig. 1). After removing duplicates, a total of 2913 articles were screened. In the subsequent stages of this process, a total of 272 full‐text reports were assessed for eligibility, of which 264 studies were excluded because the focus was not on war or peacebuilding, or not on public health education/training.

A total of seven articles reported on existing education/training or recommendation for training fulfilling the inclusion criteria (Fig. 1) [20, 32–37]. We classified the study design of the 7 articles into three groups: 4 were reports [32–34, 36]; two were commentaries [20, 37]; and one was an empirical qualitative study [35].

### Study characteristics

The 7 articles were published between 1998 and 2019, with most of them produced in the Global North, including the US (*n* = 3) [20, 32, 35], Croatia and Bosnia-Herzegovina (*n* = 1) [33] and Sweden (*n* = 1) [34]. Only one was conducted in the Global South, the empirical study from South Sudan [37]. The included publications take different phases of war into account, some take on several within one study: 4 publications reported on early warning/prevention and preparedness [33–36]; 4 publications on conflict situations [20, 33, 35, 37]; 3 on emergency [20, 32, 37]; and one study on recovery / rehabilitation [35]. Four publications related to specific armed conflicts and time periods; (Balkan (1994–2001) [32], Sweden (time period not specified) [34], South Sudan (2013) [37], and after the First World War (1920–1939) [36]. Three studies did not specify the geographical region or period of the study. The characteristics of each study are shown in Table 3, depicting the type of publication, type of war, period, geographic setting, and phase of military operations. Table 3 provides an overview of the teaching interventions recommended or described by the included studies.

**Table 3:** Study Characteristics

Author(s)	Type of publication	Type of war/armed conflict			Educational Programme			
		**Type of war**	**Geographical setting**	**Periodic reference**	**Phase of war**	**Target group**	**Level and Type of Program**	**Stage of Implementation**
**Burkle et al. (2019)** [20]	Commentary	War and armed conflict	-	Post-Cold War era	(2) Conflict & Emergency	Public Health leaders	Not specified, covering diverse types of trainings and programs	Recommendations for the development of teaching material for health care providers in war-settings (War surgeons, PH Leaders, Emergency Medical Teams)
**Evans et al. (2016)** [32]	Report	War (not further specified) and armed conflict	-	-	(2) Conflict & Emergency (3) Recovery, Rehabilitation and Peacebuilding	Public Health professionals	Standalone short-courses, selective course ranging from 2 days to 1-week, additional practical and/or research projects for graduate and post-graduate students of Public Health (for humanitarian emergencies response/education)	Established program at Emory University (Atlanta, Georgia, USA)
**Joshi (1998)** [33]	Report	Cross-border war	Balkan (Europe)	1994–2001	(1) Prevention and Preparedness (2) Conflict & Emergency	Mental Health professionals (psychologists and psychiatrists)	Professional training (not integrated in academic education)	Presentation of lessons learned
**Kulling & Holst (2003)** [34]	Report	War (not further specified)	Sweden	-	(1) Prevention and Preparedness	Interdisciplinary	Selective courses for professionals	Implemented
**McDonnell et al. (2004)** [35]	Empirical study	Acute and chronic war—further unspecified	-	-	(1) Prevention and Preparedness (2) Conflict & Emergency (3) Recovery, Rehabilitation and Peacebuilding	(Applied) Epidemiology	Not specified	Recommendation for the development of teaching material for the applied epidemiologist training programs
**McGann (2008)** [36]	Empirical study	Cross-border war (WWI, WWII)	-	1920–39	(1) Prevention and Preparedness	Nursing staff	Professional add-on training	Historical description
**Ratner & Katona (2016)** [37]	Letter to the Editor	Civil war/cross border war	South Sudan	2013	(2) Conflict & Emergency	Global Health professionals & community leaders	Multi-Day training (Wilderness First Aid Course) with lectures and firsthand sessions	Implemented

### Prevention and Preparedness

Four (*n* = 4) publications reported or commented on early warning, prevention and preparedness for war or war-like situations on a professional level. McDonnell et al. recommend that applied epidemiologists should be prepared for war by acquiring knowledge on international law, human rights, and complex interventions by working on specific case studies during training to be able to conduct assessments of the conflict setting and to communicate health-related interventions effectively with stakeholders and the local population [35]. Joshi strengthens this approach to take into account the psychological burden of mental health professionals working with war-affected children. He recommends analyzing the situation properly, gaining knowledge about the region, culture, and people, but also reflecting on one’s own limitations and resources [33]. Both commentaries point to the importance of (interdisciplinary) collaboration and training [33, 35].

Kulling & Holst and McGann have shown what a training module could look like. While Kulling & Holst refer to the Swedish context, McGann describes in a historical analysis what the training of public health nurses looked like after the First World War [34, 36]. On a national level, according to Kulling & Holst different topics should be addressed in regional and local courses for health professionals, such as disaster medicine, management of the healthcare system in a disaster/crisis, command and control at an accident site, chemical accidents, decontamination methods, radiation accidents, microbiological preparedness / bioterrorism, psychiatric / psycho-social support and planning preparedness for chemical, biological, or nuclear/radiological (C B N R) events on a national level [34]. McGann describes that the training of public health nurses in the 1920s consisted of both a theoretical and a practical part [36]. In the theoretical part there are already overlaps with the modules recommended by Kulling & Holst [34]. McGann shows that lectures were given on public health nursing, hygiene, bacteriology, psychology, social conditions, and social administration [36]. The practical part consisted of work placements in nursing-related fields, such as child welfare centers, TB dispensaries or in a district nursing association [36].

### Conflict and emergency

Burkle et al. and McDonnell et al. refer to the importance of combining medical skills and knowledge in emergency and crisis situations [20, 35]. Both reports discuss advanced courses for the development of specific skills for work in humanitarian settings. According to the authors, knowledge of legal frameworks, communication skills, documentation of human rights violations, and the design and management of needs-based health services are necessary skills for public health professionals, with McDonnell et al. also emphasizing competence in qualitative and quantiative data [20, 35]. Burkle et al. recommend developing an all-encompassing international program which should be continously evaluated and adapted according to emergency sitations [20]. Joshi adds the importance of compentency in interdisciplinary cooperation and training of local populations [33].

Evans et al. and Rathner & Katona describe specific training programs in more detail [32, 37]. While Evans et al. describe a program for graduated professionals and mid-careers public health leaders [32], Ratner & Katona’s training program focuses not only on public health professionals but also on the general public [37]. Both programs consist of lectures and hands-on-activities or practical work. Evans et al. describe a graduate program based at the Center for Humanitarian Emergencies at Emory University in Atlanta, US. The program covers topics on emergency preparedness, logistics, mental health, needs assessment, nutrition, and risk communication through lectures and group discussions, followed by a field practica at in-country host institutions. The field practica cover areas such as emergency management or global health security [32]. Ratner & Katona’s program is set in South Sudan and involves first aid courses, providing participants with the skills and knowledge to care for themselves and others in times of medical emergencies. The training brings together people from different tribes or community groups and focuses on specific medical needs. Supported by the local community and local leaders, the training leads to intergroup communication, stigma reduction, and health-related collaboration between different population groups. It not only provided essential healthcare skills, but also served as a platform for peacebuilding and community-building [37].

### Recovery, rehabilitation and peacebuilding

All authors understand their programs as preparation for war-like situations but only two focus specifically on peacebuilding [35, 37]. Ratner & Katona’s teaching activity in South Sudan lead to peacebuilding, communication, and interaction between different groups in the local communities [37]. McDonald et al. emphasize conflict assessment for peacebuilding, using quantitative and qualitative methods and effective communication skills for policy changes and interdisciplinary and interinstitutional cooperation. According to the authors, knowledge about predictors of violent conflict is necessary [35]. None of the studies in our review pertain to recovery or rehabilitation-related competencies or training programs.

## Discussion

We identified 7 publications dealing with education and/or training for public health professionals that related to war and peacebuilding [20, 32–37]. Most of the publications covered public health training from prevention and preparedness, conflict and emergency to recovery, rehabilitation and peacebuilding. Literature is scarce regarding public health education in the context of war, armed conflict and peace promotion. We found a broad range of different manuscripts including commentaries and opinion pieces on different types of war and armed conflict, but only 7 publications met the review’s inclusion criteria. We did not identify research studies comparing different teaching methods, training modules or evaluating programs. However, there is a growing awareness of the topic and not least since Russia’s invasion of Ukraine (e.g. [19, 38–40].). Nevertheless, more in-depth research needs to be done in this area.

The included publications are heterogeneous in terms of population, time, and war phases. Most of the programs described in the publications are aimed at public health professionals. Three studies (*n* = 3) focused on specific professional groups (e.g. psychologists, surgeons, nurses) [20, 33, 36]. Only Evans et al. targeted undergraduate and postgraduate students [32]. Just one publication included the local population in their education program and, in contrast to the other publications the context of the education program was within an emergency situation whereby there was an acute need for action due to the war-like situation in South Sudan [37]. Two studies described the educational program in more detail: While Kulling & Holst presented a current program in Sweden [34], McGann took a historical perspective and described the education of public health nurses between the First and Second World War [36]. Both studies pointed to similar teaching content for preparedness of professionals. Two other studies addressed general principals such as knowledge on human rights and complex interventions or self-reflection [33, 35]. None of these publications used a conceptual educational framework.

We note from our scoping review that teaching war in public health education programs or in the training of public health professionals is predominately short-term and extra-curricular in post-graduate courses. A better understanding is needed of the intersections between war and health and of the indispensable role public health practitioners, academics and advocates could play particularly given the increasing significance of war as a determinant affecting population health [31].

The immediate emergency response in times of war was the main area of action in and for public health education. Some of the studies drew from or were also closely entangled with emergency management and/or disaster management (without any specific reference to war or peacebuilding). This finding is not surprising, given the relevance of this much more advanced and established field of public health practice. Emergency response and management, including infectious disease outbreaks and/or disasters especially natural disasters, are widely included in international standards and recommendations. For example, US [41] for public health education [42]. These topics are also frequently and systematically implemented in dedicated public health degrees [43–46]. These competencies play a major role for public response in the context of war, e.g. for first response, multidisciplinary coordination and crisis situation [44]. However, we also found that emergency management and public health education on war were often entangled, which led to a high number of full-text screenings. Few of the studies and commentaries explicitly differentiated between natural disasters or outbreaks and war/armed conflicts; yet wars require additional skillsets and competencies. For example, conflicts frequently result in waves of trauma cases and public health hazards depending on conflict intensity. Also, infrastructure can be repeatedly destroyed or supplies to rebuild cannot reach communities in need – again depending on conflict activities. Accordingly, we encourage studies and commentaries on public health practice and analysis to more explicitly differentiate between natural disasters and armed conflicts [6].

Highlighting the different disciplines involved as well as the range of competencies required in different phases of war and peacebuilding, our review reiterates the importance of interdisciplinary collaboration for developing and implementing public health education on war and peacebuilding. Building on this evidence, the growing conceptual understanding [6, 14, 16, 22] and existing content-analyses of war-related public health education [20, 31] will help to systematically advance public health training as well as the scientific discourse on this topic to support evidence-based decision-making for curriculum adaptations, teaching methods as well as adaptation for peacebuilding and in times of war.

Peacebuilding and war prevention were less discussed in the publications included in this review compared to other phases of war. Whoerle et al. suggest that health education can serve as a potential platform for integrating peace education into school curricula. The integration of health and peace involves four key approaches that could be translated into competencies: adopting a socio-ecological perspective; employing complexity thinking and problem mapping, recognizing the continuum of resilience and trauma, and considering the community as a site for practical implementation, calling for interdisciplinary cooperation [16]. Barry S. Levy, one of the long-standing experts in this field, proposes that citizens should confront the powerful in their country [6]. This is important advice in democratic societies, and there are precedents of successful protest and civic disobedience such as Daniel Ellsberg’s activism against the Vietnam war, which gave a boost to the US anti-war movement [47]. We reiterate this call and encourage to focus in on the competencies in public health required to specifically include peace promotion and war prevention in addition to the emergency war response. This is particularly important in an ‘era of geopolitical uncertainty’ [48] where peace and war play a dominant role and should be reflected as determinants of health and accordingly systematically addressed in public health education [49]. However, we also realize that advice such as Levy’s is tailored more at civil wars and wars attempting regime change, rather than wars of annexation. Russia’s full-scale invasion of Ukraine in 2022, was the first such event in Europe since World War II. Internal civic action, as recommended by Levy, carries grave personal risk when applied against the Putin regime. Diplomacy has not been successful as Russia, in the eyes of many observers, violates international treaties and security assurances [50]. In consequence, neighboring states may have to rely on a sufficiently funded military to protect their populations. This conclusion may come counter-intuitive to public health proponents [51], while in defense theories and international law and relation studies it is widely discussed and elaborated on [52, 53]. Again, drawing from interdisciplinary collaboration could ensure deeper analytical and theoretical understandings of the terminology or concepts used in war and peacebuilding and comparability across fields. Moreover, given the contentious nature of these issues, students and teaching institutions should learn how to discuss conflicts constructively and fairly, avoiding the escalations of recent campus discourses in the Israel-Gaza conflict.

### Strengths and limitations

In this Scoping Review, we analyzed the scope and extent of scientific discourse on war and peace promotion in public health education. Using a scoping review methodology allowed for a systematized and comprehensive mapping, which has been the first of its kind on the topic, at least to our knowledge. We included studies solely with a primary interest in war and peacebuilding in the public health education context. This allowed for a nuanced analysis of education programs, initiatives, or similar, which is of particular value for the overall aim of advancing public health education.

We thereby contribute to the identification of gaps and addressing them in an important area of public health work. Specifically, we have identified gaps in the European literature arena, where the topic of war and peace promotion has been mostly neglected over the past decades. In addition, we have synthesized war and peace promotion in public health education, which are usually assessed in parallel.

We limited our review to scientific databases aiming to assess the scientific discourse on war and peacebuilding in public health education and training. As a result, the review has not captured any grey literature relating to the topic; it exceeded the scope of this review. Nonetheless, it would be an important next step to assess the status quo at institutions of public health education and training within the European region. This includes Schools of Public Health and Higher Institutes or Centers for Public Health Education, many of whom lead to a graduate degree in public health and which is accredited by a recognized body, or bodies approved for such purpose. This could include for example the Secretary of Education in each European Region country or other authorizing bodies. Of note, a study in this regard, has been undertaken for specific schools of public health in the United States of America [31]. Such a study in Europe would allow for a more detailed overview of resources and expertise to advance and support the development of initiatives integrate war more systematically into public health education programs.

Since we only included studies with a primary interest in war and peacebuilding in the public health education context, we did not consider the scientific discussion touching on this topic. This was reflected many of full texts screened that ended with just a few publications with findings that could be generalized. The variability in the studies in terms of the type of war described, e.g., armed conflict or war of annexation, may present a challenge in drawing generalizable conclusions.

## Conclusion

This scoping review identified a lack of scientific discourse on the role of war and peace promotion in public health education. The few public health education and training programs identified primarily focus on the immediate emergency response in conflict-affected areas. These were often entangled with emergency preparedness in different contexts such as infectious disease outbreaks. Peace promotion and war prevention are missing foci. This suggests a need for a far greater emphasis of the topic in public health training as well as its inclusion in competency frameworks. In addition to training public health professionals for emergencies, training with an emphasis on war prevention and promoting peace should be developed and implemented.

### Supplementary Information

Supplementary Material 1.

Supplementary Material 2.

Supplementary Material 3.

## Data Availability

All data generated or analyzed during this study are included in this published article [and its supplementary information files].
