# Supplementary material for: War and peace in public health education and training: a scoping review
Source: BMC Public Health. 2024 Aug 24;24:2303. doi: 10.1186/s12889-024-19788-w (PMC11344335; doi:10.1186/s12889-024-19788-w)
Supplement: Supplementary file 2 — Supplementary Material 2. [file 12889_2024_19788_MOESM2_ESM.docx]

**Additional file 2: Search protocols**

Searches conducted on 08.09.2022

## **PubMed**

("teaching"[Title/Abstract] OR "education"[Title/Abstract] OR "training"[Title/Abstract] OR "course"[Title/Abstract] OR "classroom"[Title/Abstract] OR "workforce development"[Title/Abstract] OR "capacity building"[Title/Abstract] OR "competence"[Title/Abstract] OR "competencies"[Title/Abstract] OR "curriculum"[Title/Abstract] OR "curricula"[Title/Abstract] OR "syllabus"[Title/Abstract] OR "syllabi"[Title/Abstract] OR "pedagogy"[Title/Abstract] OR "pedagogic"[Title/Abstract] OR "toolkit"[Title/Abstract] OR "schools of public health"[Title/Abstract] OR "school of public health"[Title/Abstract] OR "public health department"[Title/Abstract] OR "public health faculty"[Title/Abstract]) AND ("war"[Title/Abstract] OR "armed conflict"[Title/Abstract] OR "mass violence"[Title/Abstract] OR "warfare"[Title/Abstract] OR "combat"[Title/Abstract] OR "military"[Title/Abstract] OR "peace"[Title/Abstract] OR "peace promotion"[Title/Abstract] OR "peace-building"[Title/Abstract] OR "peace-building"[Title/Abstract] OR "disaster management"[Title/Abstract] OR "emergency response"[Title/Abstract] OR "emergency preparedness"[Title/Abstract] OR "conflict response"[Title/Abstract] OR "disaster recovery"[Title/Abstract] OR "humanitarian crisis"[Title/Abstract]) AND ("public health"[Title/Abstract] OR "global health"[Title/Abstract])

## **Embase**

('teaching':ab,ti OR 'education':ab,ti OR 'training':ab,ti OR 'course':ab,ti OR 'classroom':ab,ti OR 'workforce development':ab,ti OR 'capacity building':ab,ti OR 'competence':ab,ti OR 'competencies':ab,ti OR 'curriculum':ab,ti OR 'curricula':ab,ti OR syllabus:ab,ti OR syllabi:ab,ti OR pedagogy:ab,ti OR pedagogic:ab,ti OR 'toolkit':ab,ti OR 'schools of public health':ab,ti OR 'school of public health':ab,ti OR 'public health department':ab,ti OR 'public health faculty':ab,ti) AND ('war':ab,ti OR 'armed conflict':ab,ti OR 'mass violence':ab,ti OR warfare:ab,ti OR combat:ab,ti OR 'military':ab,ti OR peace:ab,ti OR 'peace promotion':ab,ti OR 'peace-building':ab,ti OR 'peace building':ab,ti OR 'disaster management':ab,ti OR 'emergency response':ab,ti OR 'emergency preparedness':ab,ti OR 'conflict response':ab,ti OR 'disaster recovery':ab,ti OR 'humanitarian crisis':ab,ti) AND ('public health':ab,ti OR 'global health':ab,ti) AND ([embase]/lim NOT ([embase]/lim AND [medline]/lim))

## **CINAHL**

AB ( “teaching” OR “education” OR “training” OR “course” OR “classroom” OR “workforce development” OR “capacity building” OR “competence” OR “competencies” OR “curriculum” OR “curricula” OR „syllabus“ OR “syllabi“ OR “pedagogy“ OR “pedagogic“ OR “toolkit” OR “schools of public health” OR “school of public health“ OR “public health department” OR “public health faculty” ) AND AB ( “war” OR “armed conflict” OR “mass violence” OR “warfare“ OR “combat“ OR “military” OR “peace“ OR “peace promotion” OR “peace-building” OR “peace building” OR “disaster management” OR “emergency response” OR “emergency preparedness” OR “conflict response“ OR “disaster recovery” OR “humanitarian crisis” ) AND AB ( “public health” OR “global health” )

## **PsycINFO**

AB ( “teaching” OR “education” OR “training” OR “course” OR “classroom” OR “workforce development” OR “capacity building” OR “competence” OR “competencies” OR “curriculum” OR “curricula” OR „syllabus“ OR “syllabi“ OR “pedagogy“ OR “pedagogic“ OR “toolkit” OR “schools of public health” OR “school of public health“ OR “public health department” OR “public health faculty” ) AND AB ( “war” OR “armed conflict” OR “mass violence” OR “warfare“ OR “combat“ OR “military” OR “peace“ OR “peace promotion” OR “peace-building” OR “peace building” OR “disaster management” OR “emergency response” OR “emergency preparedness” OR “conflict response“ OR “disaster recovery” OR “humanitarian crisis” ) AND AB ( “public health” OR “global health” )

## **Web of Science Core Collection**

((AB=(“public health” OR “global health”)) AND AB=(“war” OR “armed conflict” OR “mass violence” OR “warfare“ OR “combat“ OR “military” OR “peace“ OR “peace promotion” OR “peace-building” OR “peace building” OR “disaster management” OR “emergency response” OR “emergency preparedness” OR “conflict response“ OR “disaster recovery” OR “humanitarian crisis”)) AND AB=(“teaching” OR “education” OR “training” OR “course” OR “classroom” OR “workforce development” OR “capacity building” OR “competence” OR “competencies” OR “curriculum” OR “curricula” OR "syllabus“ OR “syllabi“ OR “pedagogy“ OR “pedagogic“ OR “toolkit” OR “schools of public health” OR “school of public health“ OR “public health department” OR “public health faculty”)

Query link

<https://www.webofscience.com/wos/woscc/summary/6caad901-9cb0-442f-b169-ab2bc4ac79fc-4da6bd72/relevance/1>
