# Supplementary material for: War and peace in public health education and training: a scoping review
Source: BMC Public Health. 2024 Aug 24;24:2303. doi: 10.1186/s12889-024-19788-w (PMC11344335; doi:10.1186/s12889-024-19788-w)
Supplement: Supplementary file 3 — Supplementary Material 3. [file 12889_2024_19788_MOESM3_ESM.docx]

# War and peace in public health education: a Scoping Review

# Review protocol

## Rationale & aims

Aim: Map the available evidence and discourse on war, including war prevention, reaction to war as well as rehabilitation, and peace promotion in the public health education and training context, as reflected in the scientific literature.

Research questions:

- To what extent and how is public health education addressing competencies related to war?
- Can we build future training efforts on good practice examples of teaching?
- What are the gaps and blind spots in public health education on war?

Added value of conducting the review: global outlook (not limited to US context), nexus of public health research and teaching – how are these two linked and inform each other?, how is the workforce development supported by public health research.

Table 1 PCC scheme to guide the research question

| Population | Public health higher education: from the institutions/schools engaged in teaching to school-level programmes (including bachelors, masters and PhD programmes) and individual classes/courses on a global scale |
| --- | --- |
| Concept | War, armed conflict - their impact on public health as well as the public health response/reaction to war & armed conflict  Prevention of war and peace-building work  (Emergency response, disaster management) |
| Context | Discipline of public health |

# Methods

Scoping review with a narrative synthesis (using the PRISMA Scoping Review Checklist)

SR characteristics that might be beneficial for our research interest:

- Aims to identify the “nature and extent of evidence” (Grant & Booth, 2009)
- Allows for a “broader” but still comprehensive and systematic approach
- Allows to map a diverse range of evidence e.g. implementation research on training programmes as well as commentaries & opinion pieces as indicators of an ongoing discourse within the public health community

## Search strategy

Scientific papers in academic databases*:* PubMed, CINAHL, PsycINFO, Embase, Web of Science Core Collections

Table 2 Search strategy in PubMed

| **PCC element** (linked by AND) | **Search string** |
| --- | --- |
| **Population** | “teaching” OR “education” OR “training” OR “course” OR “classroom” OR “workforce development” OR “capacity building” OR “competence” OR “competencies” OR “curriculum” OR “curricula” OR „syllabus“ OR “syllabi“ OR “pedagogy“ OR “pedagogic“ OR “toolkit” OR “schools of public health” OR “school of public health“ OR “public health department” OR “public health faculty” |
| **Concept** | “war” OR “armed conflict” OR “mass violence” OR “warfare“ OR “combat“ OR “military” OR  “peace“ OR “peace promotion” OR “peace-building” OR “peace building” OR  “disaster management” OR “emergency response” OR “emergency preparedness” OR “conflict response“ OR “disaster recovery” OR “humanitarian crisis” |
| **Context** | “public health” OR “global health” |

Filter: Title/Abstract

🡪 in a lot of work public health education is identified as one of the fields where action is needed, but it does not represent the major interest of the study (which is an important issue as well, but adds a lot of “noise” to the search and significantly extends the number of records substantially)

*Please see at the end of the document for overview of different combinations of the searchers*

In addition to the search in scientific databases, we check reference lists and citations of included material

## Inclusion & exclusion criteria

Table 3 Inclusion and exclusion criteria

| **Inclusion** | **Exclusion** |
| --- | --- |
| - The main interest is on war and peace in the public health education context. This can include the development of competencies and capacity building as well as the structural context of the bodies providing public health education. - Academic literature (incl. commentaries, editorials, opinion pieces, conference abstracts to describe the discourse within the field) - No exclusion based on time or language | - The target group of training & capacity development is not within the public health workforce - Programmes/courses/classes/ institutions outside of public health |

Screening process:

- At least two reviewers (LW, CC) conduct the screening, no duplicate screening
- Do a pilot screening to increase inter-rater reliability (about 5-10% of the final records)
- Start with title/abstract screening, continue with full-text screening, final inclusion of full-texts will be checked by all reviewers

## Data extraction

Table 4 Data extraction template

| **Category** | **Description** |
| --- | --- |
| Reference | Full reference of the original article |
| Type of material | Study, commentary/editorial, dissertation, book chapter |
| Study design | Qualitative, quantitative or mixed-methods study, plus more detailed information on the study (e.g. cross-sectional study, focus groups) |
| Geographic origin of the material | For studies: the country/countries where the study has been conducted  For commentaries/opinion pieces: list all countries identified by the authors’ affiliations |
| Key topic | Overarching topics might include   - Health care system level - Legal dimension of war (international conventions, law, types of war) - “vulnerable” populations (children, women, LGBTQI*, displaced people, detainees, veterans) - Prevention of war & Peace building work |
| Discipline of public health | e.g. epidemiology, global health, public health law  target group |
| Aims | Aims as stated by the authors |
| Main findings // arguments | For studies: what are the main findings?  For opinion pieces: what is the main argument of the authors? (e.g. what do they call for? What kind of gap have they identified? What would be the next steps?) |
| **Teaching material** |  |
| Brief description of training | What is this training about? How has this been developed? Who was part of the process developing and implementing it? |
| Level of programme | Bachelors, Masters, PhD |
| Type of programme | Integration in curricula or add-on material? Mandatory or selective courses? |
| Competencies covered in the material | Which competences does the teaching material address?  🡪 Guided by the WHO-ASPHER competency framework that we intend to apply when developing the ASPHER curriculum  Content and context   1. Science and practice 2. Promoting health 3. Law, policies, ethics 4. One Health and health security   Relations and interactions   1. Leadership and systems thinking 2. Collaboration and partnerships 3. Communication, culture and advocacy   Performance and achievement   1. Governance and resource management 2. Professional development and reflective ethical practice 3. Organizational literacy and adaptability |
| Level of development | Development or presentation of teaching material, implementation, or evaluation research? |
| Availability of the material | Is the teaching material publicly available? |

Data extraction process:

- Also do a pilot for the data extraction to increase inter-rater reliability
- Data extraction in a tabular form (Excel file)

## Synthesis

Descriptive, narrative synthesis of findings

**Development of the search strategy**

Preferred search now:

Complete inclusion of search terms:

Search: **(("teaching"[Title/Abstract] OR "education"[Title/Abstract] OR "training"[Title/Abstract] OR "course"[Title/Abstract] OR "classroom"[Title/Abstract] OR "workforce development"[Title/Abstract] OR "capacity building"[Title/Abstract] OR "competence"[Title/Abstract] OR "competencies"[Title/Abstract] OR "curriculum"[Title/Abstract] OR "curricula"[Title/Abstract] OR „syllabus"[Title/Abstract] OR „syllabi"[Title/Abstract] OR „pedagogy"[Title/Abstract] OR „pedagogic"[Title/Abstract] OR "toolkit"[Title/Abstract] OR "schools of public health"[Title/Abstract] OR „school of public health"[Title/Abstract] OR "public health department"[Title/Abstract] OR "public health faculty"[Title/Abstract]) AND ("war"[Title/Abstract] OR "armed conflict"[Title/Abstract] OR "mass violence"[Title/Abstract] OR „warfare"[Title/Abstract] OR „combat"[Title/Abstract] OR "military"[Title/Abstract] OR „peace"[Title/Abstract] OR "peace promotion"[Title/Abstract] OR "peace-building"[Title/Abstract] OR "peace building"[Title/Abstract])) AND ("public health"[Title/Abstract] OR "global health"[Title/Abstract])**

("teaching"[Title/Abstract] OR "education"[Title/Abstract] OR "training"[Title/Abstract] OR "course"[Title/Abstract] OR "classroom"[Title/Abstract] OR "workforce development"[Title/Abstract] OR "capacity building"[Title/Abstract] OR "competence"[Title/Abstract] OR "competencies"[Title/Abstract] OR "curriculum"[Title/Abstract] OR "curricula"[Title/Abstract] OR "syllabus"[Title/Abstract] OR "syllabi"[Title/Abstract] OR "pedagogy"[Title/Abstract] OR "pedagogic"[Title/Abstract] OR "toolkit"[Title/Abstract] OR "schools of public health"[Title/Abstract] OR "school of public health"[Title/Abstract] OR "public health department"[Title/Abstract] OR "public health faculty"[Title/Abstract]) AND ("war"[Title/Abstract] OR "armed conflict"[Title/Abstract] OR "mass violence"[Title/Abstract] OR "warfare"[Title/Abstract] OR "combat"[Title/Abstract] OR "military"[Title/Abstract] OR "peace"[Title/Abstract] OR "peace promotion"[Title/Abstract] OR "peace-building"[Title/Abstract] OR "peace-building"[Title/Abstract]) AND ("public health"[Title/Abstract] OR "global health"[Title/Abstract])

N=1,283

(without global health 1,181 results)

Search in PubMed:

Search: **(("teaching"[Title/Abstract] OR "education"[Title/Abstract] OR "training"[Title/Abstract] OR "course"[Title/Abstract] OR "classroom"[Title/Abstract] OR "workforce development"[Title/Abstract] OR "capacity building"[Title/Abstract] OR "competence"[Title/Abstract] OR "competencies"[Title/Abstract] OR "curriculum"[Title/Abstract] OR "curricula"[Title/Abstract] OR „syllabus"[Title/Abstract] OR „syllabi"[Title/Abstract] OR „pedagogy"[Title/Abstract] OR „pedagogic"[Title/Abstract] OR "toolkit"[Title/Abstract] OR "schools of public health"[Title/Abstract] OR „school of public health"[Title/Abstract] OR "public health department"[Title/Abstract] OR "public health faculty"[Title/Abstract]) AND ("war"[Title/Abstract] OR "armed conflict"[Title/Abstract] OR "mass violence"[Title/Abstract] OR „warfare"[Title/Abstract] OR „combat"[Title/Abstract] OR "military"[Title/Abstract] OR „peace"[Title/Abstract] OR "peace promotion"[Title/Abstract] OR "peace-building"[Title/Abstract] OR "peace building"[Title/Abstract] OR "disaster management"[Title/Abstract] OR "emergency response"[Title/Abstract] OR "emergency preparedness"[Title/Abstract] OR „conflict response"[Title/Abstract] OR "disaster recovery"[Title/Abstract])) AND ("public health"[Title/Abstract] OR "global health"[Title/Abstract])**

N=1,908

(without global health 1,786 results)
